# Association of the STAT3 rs1053004 Polymorphism With Hepatocellular Carcinoma in Iraqi Patients With Hepatitis B

**DOI:** 10.1155/ijh/6122232

**Published:** 2026-07-29

**Authors:** Hawraa Ahmed Ali, Thuraya Aamer Habeeb, Zahraa F. Shaker, Zahraa Q. Jasim

**Affiliations:** ^1^ Department of Pharmacy Techniques, Babylon Technical Institute, Al-Furat Al-Awsat Technical University, Al-Najaf, Iraq, atu.edu.iq; ^2^ Department of Medical Laboratory Techniques, Polytechnic College-Karbala, Al-Furat Al-Awsat Technical University, Al-Najaf, Iraq, atu.edu.iq; ^3^ College of Dentistry, Mustansiriyah University, Baghdad, Iraq, uomustansiriyah.edu.iq

**Keywords:** hepatitis B virus, hepatocellular carcinoma, Iraq, single-nucleotide polymorphism, STAT3

## Abstract

**Introduction:**

HCC is still one of the main causes of cancer‐related fatalities despite years of intensive attempts to prevent it. One known risk factor is a persistent hepatitis B virus (HBV) infection. Previous studies have indicated a contribution of the STAT3 signaling pathway to carcinogenesis through inflammation mechanisms. In this study, we tried to explore the correlation between STAT3 rs1053004 polymorphism and HCC occurrence in people with HBV infection in Iraq.

**Methods:**

A case–control approach was applied with the inclusion of 80 patients with HCC due to HBV infection, 80 patients with CHB, and 80 healthy controls. The study was based on genotyping of the STAT3 rs1053004 polymorphism by a TaqMan SNP genotyping assay. Multivariate logistic regression analysis was used to assess the genetic association with disease risk, adjusting for potential confounding factors.

**Results:**

The three groups under study had significantly different genotype and allele distributions (*p* < 0.001). The HCC group had more T alleles than the other groups. Additionally, patients with the TT genotype had a greater risk of HCC (adjusted odds ratio [AOR] = 4.26; 95*%*confidence interval [CI] = 1.89–9.62; *p* < 0.001), and this impact was observed in patients with cirrhosis, high serum HBV DNA levels, and negative HBeAg status.

**Conclusion:**

STAT3 rs1053004 polymorphism plays a significant role in the development of HCC risk in people with HBV infection.

## 1. Introduction

In 2023, hepatocellular carcinoma (HCC) had about 906,000 new cases and 830,000 cancer deaths, thus making HCC the sixth commonest type of cancer across the world and the third cause of cancer mortality [1]. One of the risk factors for HCC includes hepatitis B virus (HBV) infection, which is very common in regions where this cancer is common [2]. In spite of many vaccination programs against HCC, HBV infection remains a significant health problem in Iraq due to low to moderate prevalence levels [3]. The development of HCC from a chronic HBV infection is complex and involves the influence of both the virus itself and other factors, such as the environment and the patient′s genotype [4]. Recent developments in genomic research have shown that genetic modifications are responsible for influencing the likelihood of developing HCC. Certain single‐nucleotide polymorphisms (SNPs) related to inflammation and cell proliferation processes have been identified as predictive markers for the disease among humans [5]. According to recent researches, STAT3, which plays an important part in the development of HCC, is one of the key factors in this sphere. The activation of STAT3 results in increased proliferation of tumor cells and decreased apoptosis and resistance to immunological mechanisms of elimination; therefore, it usually has unfavorable prognostic significance [6, 7]. Besides, STAT3 is the central element of the JAK/STAT signaling pathway, regulating the cellular response to cytokines (especially IL‐6) and other growth factors [8, 9]. Constitutive activation of STAT3 has been observed in different types of cancers such as HCC and has also shown to contribute to tumor growth, survival, angiogenesis, and evasion of immunity [7]. The rs1053004 polymorphism is present in the 3 ^′^‐untranslated region of the *STAT3* gene and regulates the expression and function of the *STAT3* gene. Various research articles have analyzed the relationship between different polymorphisms in the *STAT3* gene and cancer risk [10]. A meta‐analysis study has established a strong connection between certain polymorphisms in the *STAT3* gene and cancer risk [11]. On the other hand, very few studies have explored the connection between the STAT3 rs1053004 SNP and the risk of HCC among HBV‐infected individuals [10, 12, 13]. According to Fatemipour et al. [12], a case‐control study found a correlation between STAT3 mutations and the occurrence of HCC among Asian individuals. According to Xie et al. [14], however, there may be some interactions between the status of HBV infection and specific genetic mutations in the *STAT3* gene. Recent studies carried out in Iraq have been largely focused on the epidemiology and clinic features of HBV infection and HCC. There is a lack of studies investigating genetic risk factors. Currently existing research findings also emphasize the requirement for further examination of genetic and molecular markers and noninvasive methods of diagnosis of HCC and related liver diseases caused by HBV among Iraqi populations [3, 15, 16]. The study by Habeeb et al. [17] emphasized the need to conduct further research on liver cirrhosis and HCC linked to HBV infections in Iraq. The research project attempts to fill an identified gap within the existing body of knowledge since previous researches did not investigate the effect of *STAT3* gene polymorphism on the risk of developing HCC in patients from the current study population. An interesting knowledge gap exists in the context that gene associations vary greatly depending on ethnicity due to the influence of host genetics, environment, and viruses such as HBV. The goal of the research is to examine the impact of the rs1053004 gene variant of STAT3 on the risk of HCC among Iraqi patients infected with HBV. In this regard, the research will try to establish a role for STAT3 gene polymorphism on the prediction of HCC risk. Ultimately, the proposed study will advance towards a personalized HCC risk predictor for Iraqi patients with chronic HBV infection.

## 2. Materials and Methodology

### 2.1. Participants and Study Design

This study was done in AL‐Hussaini Teaching Hospital in Karbala, Iraq, from December 2024 to June 2025. Permission was given by the Research Management Unit of the Karbala Health Directorate, Iraqi Ministry of Health, for the conduct of this experiment (Approval Number: 309, dated February 19, 2025). This study followed ethical principles set forth in the Helsinki Declaration along with its amendments. Ethical permission was given by the research ethics committee for human participation and informed consent. Each patient signed informed consent forms. Spoken informed consent was taken from those who could not read. It was done in front of an independent witness.

A total of 240 patients were used in this experiment, and these patients were divided into three groups:

1. HCC in individuals with HBV infection (*n* = 80). Dynamic magnetic resonance imaging (MRI) and/or multiphasic contrast‐enhanced computed tomography (CT) were performed on all individuals suspected of having HCC. The presence of distinctive imaging features, such as hyperenhancement in the arterial phase followed by washout during the portal venous and/or delayed phases, was used to diagnose HCC in compliance with the American Association for the Study of Liver Diseases (AASLD) criteria. Histopathological confirmation was sought in atypical imaging cases only. The serum level of alpha‐fetoprotein (AFP) was considered supportive information but not used as a separate criterion in the diagnosis of HCC. Nodules that were either regenerative or dysplastic were distinguished from HCC based on the lack of characteristic HCC vascular enhancement/washout imaging findings. In total, 71 patients had HCC diagnosis confirmed via imaging findings only, whereas nine patients underwent histopathology because of atypical imaging findings.
2. Chronic hepatitis B group (CHB group; *n* = 80): Subjects with CHB without clinical evidence of HCC can be characterized by elevated serum ALT levels and persistent positivity of HBsAg for over 6 months.
3. Normal control group (*n* = 80): individuals with normal results of liver function tests and absence of HBV and hepatitis C virus infection.

Exclusion criteria included any patients with autoimmune liver disease, alcoholic liver disease, nonalcoholic steatohepatitis, hemochromatosis, Wilson′s disease, coinfection with HCV or HIV, and previous cancer.

### 2.2. Clinical and Laboratory Assessments

Data related to the demographics and clinical characteristics of the patients were obtained using structured questionnaires, which were later verified by reviewing the case file of the patients. Fasting blood samples (10 mL) were taken after a 10‐ to12‐h fasting period. The tests performed on these blood samples were a complete blood count, liver function test (alanine transaminase, aspartate transaminase, total bilirubin, and albumin), kidney function tests (urea and creatinine), and AFP test. Levels of markers of HBV infections, that is, HBsAg, HBeAg, and anti‐HBe, were tested using Abbott ARCHITECT CMIA (chemiluminescent microparticle immunoassay); more specifically, HBsAg Qualitative II Reagent (Catalog No. 2G22‐25), HBeAg Reagent (Catalog No. 6C32‐25), and Anti‐HBe Reagent (Catalog No. 6C34‐25) (Abbott GmbH, Wiesbaden, Germany). Quantification of HBV DNA was done using the COBAS AmpliPrep/COBAS TaqMan HBV Test v2.0 (Roche Diagnostics, Basel, Switzerland), with a detection limit of 20 IU/mL.

### 2.3. Extraction of DNA and Genotyping

The genomic DNA was extracted from the peripheral blood leukocytes using the QIAamp DNA Blood Mini Kit (Product Code: 51104; Qiagen, Hilden, Germany) according to the manufacturer′s protocol. The concentration and purity of the genomic DNA were estimated using Nanodrop spectrophotometer (Thermo Fisher Scientific, Waltham, Massachusetts, United States). All the samples that had an A260/A280 ratio between 1.8 and 2.0 were suitable for the study. The polymorphism located on locus rs1053004 in the *STAT3* gene was genotyped by TaqMan SNP Genotyping Assay (Product ID: C__1795285_1_, Product Code: 4351379; Applied Biosystems, Thermo Fisher Scientific, Waltham, Massachusetts, United States). The genomic location of rs1053004 within the 3 ^′^‐untranslated region of the *STAT3* gene is shown in Figure S1. The procedure was conducted according to the recommendations of the manufacturer in a 20‐*μ*L reaction mixture containing 10 *μ*L of 2× TaqMan Genotyping Master Mix, 1 *μ*L of 20× TaqMan SNP Genotyping experiment, 4 *μ*L of genomic DNA (5 ng/*μ*L), and 5 *μ*L of nuclease‐free water. The procedure of polymerase chain reaction (PCR) included denaturation at 95°C for 10 min followed by 40 cycles including 15 s at 95°C (denaturation), and 60 s at 60°C (annealing‐extension). Individuals′ genotype was determined using QuantStudio Design and Analysis Software (Version 2.4; Applied Biosystems). In order to confirm our results, approximately 10% of randomly chosen samples were regenotyped by ARMS‐PCR. A 100% concordance was found between two approaches.

### 2.4. Allele‐Specific PCR (ARMS‐PCR)

• Common reverse primer: 5 ^′^‐GCCACCTCCCTCTCCCTC‐3 ^′^
• T allele–specific forward primer: 5 ^′^‐TGGGAGTAGAGCTGGAGAT‐3 ^′^ (last base = T), (120 bp).
• C allele–specific forward primer: 5 ^′^‐TGGGAGTAGAGCTGGAGAC‐3 ^′^ (last base = C), (120 bp).
• Control primer pair (internal control): (220 bp).
  o. Forward: 5 ^′^‐CTGGGAGCAGAGCTGGAGA‐3 ^′^
  o. Reverse: 5 ^′^‐GCCACCTCCCTCTCCCTC‐3 ^′^

The allele‐specific ARMS‐PCR primer design, expected amplification products, and in silico evaluation of primer characteristics are provided in Figures S2 and S3. Detailed information on primer sequences, physicochemical properties, expected amplicon sizes, allele specificity, secondary‐structure analysis, and Primer‐BLAST specificity is available in Tables S1–S4.

### 2.5. Analytical Statistics

All statistical analyses were conducted utilizing IBM SPSS 27.0 software (IBM Corp., Armonk, New York, United States) and R 4.2.2 software (R Foundation for Statistical Computing, Vienna, Austria). Quantitative variables were characterized by their means ± standard deviation (SD) or median with interquartile range (IQR), depending on their distributions, and were analyzed using Student′s *t*‐test, one‐way analysis of variance (ANOVA), Mann–Whitney *U* test, or Kruskal–Wallis test as applicable. Qualitative data were expressed as frequencies with percentages and analyzed using the chi‐square test or Fisher′s exact test. Hardy–Weinberg equilibrium (HWE) in the control group was assessed using the chi‐square goodness‐of‐fit test. The odds ratio (OR) and 95% CI of the relation between the STAT3 rs1053004 genotype and the HCC risk were calculated by using logistic regression analysis adjusting for the potential confounding factors of age, sex, smoking, and liver cirrhosis. Association analysis of the HCC‐susceptibility locus was carried out using the genetic models of codominance, dominance, recessiveness, and allelic association, which are frequently used in genetic association studies since the best model of inheritance of STAT3 rs1053004 polymorphism is still unknown. It is important to note that the interpretation of the relations was conservative because of the possible increase of Type I error in case multiple inheritance‐model tests were run. Post hoc statistical power analysis was done by applying G ^∗^Power v. Three.1 software (Heinrich Heine University Düsseldorf, Germany). Considering the effect size of 2.23 for the relation between the two main groups, that is, HCC patients and controls (*n* = 160), the study had an acceptable power (99.6%).

## 3. Results

### 3.1. Demographics and Clinical Characteristics of Study Participants

Table 1 shows the clinical and demographic features of the study participants. The average age of the study participants was statistically higher in the HCC group (59.7 ± 8.4 years) than the CHB group (48.2 ± 10.3 years) and control (45.6 ± 9.7 years) groups, *p* < 0.001. The male to female ratio of all the three groups under consideration had more males than females. However, while the male to female ratio for the three different populations analyzed showed a greater number of males than females, the male to female ratio for the HCC population proved to be higher compared with the CHB population and control population. The male to female ratio for the HCC population was 2.8:1, whereas the male to female ratio for the CHB population and control group was 1.9:1 and 1.5:1, respectively (*p* = 0.038). There was no statistically significant difference between the body mass index values of the study participants in the three groups (*p* = 0.274). A considerable percentage of patients in the HCC group had liver cirrhosis (78.8%) as compared with the CHB group (32.5%), *p* < 0.001. The positive rate of HBeAg in the HCC group was 33.8% and in the CHB group was 28.8%, *p* = 0.491. The median HBV DNA load in the HCC group was statistically higher than that of the CHB group and stood at 4.7 × 10^5^ versus 2.3 × 10^4^ IU/mL, respectively (*p* < 0.001). The serum levels of AFP in patients from the HCC group were significantly high at 328.5 ng/mL than the CHB group (6.2 ng/mL) and control group (3.4 ng/mL), *p* < 0.001. The values of liver function tests, including ALT, AST, total bilirubin, and albumin, showed significant differences among the three groups, *p* < 0.001 for each pair of comparison. The distributions of age, BMI, total bilirubin, and serum albumin across the study groups are shown in Figure S5. In addition, the distributions of AFP, HBV DNA, ALT, and AST across the study groups are presented in Figure S6.

**Table 1:** Clinical and demographic features of study participants.

Characteristic	HCC group (*n* = 80)	CHB group (*n* = 80)	Control group (*n* = 80)	*p*
Age (years), mean ± SD	59.7 ± 8.4	48.2 ± 10.3	45.6 ± 9.7	< 0.001
Sex, *n* (%)				0.038
Male	59 (73.8)	53 (66.3)	48 (60.0)	
Female	21 (26.2)	27 (33.7)	32 (40.0)	
BMI (kg/m^2^), mean ± SD	26.8 ± 4.3	27.4 ± 3.9	26.3 ± 4.1	0.274
Smoking status, *n* (%)				0.023
Current smoker	32 (40.0)	24 (30.0)	18 (22.5)	
Former smoker	18 (22.5)	16 (20.0)	15 (18.8)	
Never smoker	30 (37.5)	40 (50.0)	47 (58.7)	
Family history of HCC, *n* (%)	13 (16.3)	6 (7.5)	2 (2.5)	0.006
Liver cirrhosis, *n* (%)	63 (78.8)	26 (32.5)	—	< 0.001
Child–Pugh class, *n* (%)				< 0.001
A	29 (36.2)	16 (20.0)	—	
B	41 (51.3)	8 (10.0)	—	
C	10 (12.5)	2 (2.5)	—	
HBeAg positive, *n* (%)	27 (33.8)	23 (28.8)	—	0.491
HBV DNA (IU/mL), median (IQR)	4.7 × 10^5^ (2.3 × 10^4^–8.6 × 10^6^)	2.3 × 10^4^ (1.8 × 10^3^–3.4 × 10^5^)	—	< 0.001
AFP (ng/mL), median (IQR)	328.5 (96.7–1243.6)	6.2 (3.8–11.5)	3.4 (2.1–5.7)	< 0.001
ALT (U/L), median (IQR)	78.5 (54.2–126.8)	65.3 (42.1–98.7)	24.7 (18.5–32.6)	< 0.001
AST (U/L), median (IQR)	98.7 (67.3–156.4)	58.2 (38.6–89.4)	22.9 (17.3–29.5)	< 0.001
Total bilirubin (mg/dL), mean ± SD	2.8 ± 1.6	1.5 ± 0.9	0.8 ± 0.3	< 0.001
Albumin (g/dL), mean ± SD	3.1 ± 0.6	3.7 ± 0.5	4.2 ± 0.4	< 0.001

Abbreviations: AFP, alpha‐fetoprotein; ALT, alanine aminotransferase; AST, aspartate aminotransferase; BMI, body mass index; CHB, chronic hepatitis B; HBeAg, hepatitis B e antigen; HBV, hepatitis B virus; HCC, hepatocellular carcinoma; IQR, interquartile range; SD, standard deviation.

### 3.2. Genotype and Allele Frequencies of the STAT3 rs1053004 Polymorphism

**Figure 1:**
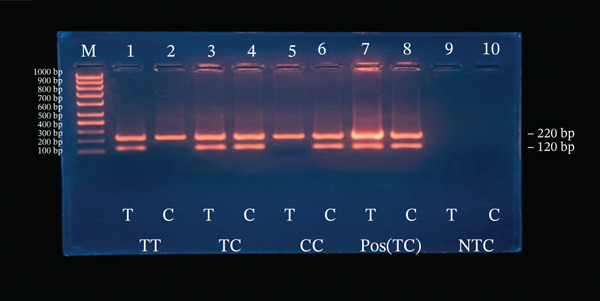
ARMS‐PCR genotyping of STAT3 rs1053004 polymorphism. Ladder Lane M: 100 bp DNA ladder. Lanes 1 and 2: TT genotype (T and C reactions). Lanes 3 and 4: TC genotype. Lanes 5 and 6: CC genotype. Lanes 7 and 8: positive control (TC). Lanes 9 and 10: negative control (NTC). Size of allele specific amplicon: 120 bp.

Table 2 shows the genotypes and alleles frequency distribution of the STAT3 rs1053004 polymorphism in the three groups under investigation; representative ARMS‐PCR genotyping results are presented in Figure 1. The genotype distribution in the control group was consistent with HWE (*p* = 0.752). There are significant differences in genotypes among the three groups (*p* < 0.001). Genotype distribution for the HCC group includes 48.8% TT, 38.8% CT, and 12.4% CC; for the CHB group comprises 35% TT, 45% CT, and 20% CC; whereas, for the control group, the frequencies include 25% TT, 47.5% CT, and 27.5% CC. The allele frequency distribution in the HCC group is significantly increased in T allele frequency as opposed to that of CHB and control groups (68.1%), which differs from the frequencies for both CHB and control groups (*p* < 0.001). On the contrary, the C allele frequency is lower in the HCC group than in CHB and control groups.

**Table 2:** Genotype and allele frequencies of STAT3 rs1053004 polymorphism in study groups.

Genotype/allele	HCC group (*n* = 80)	CHB group (*n* = 80)	Control group (*n* = 80)	*p*
Allele, *n* (%)				< 0.001
T	109 (68.1)	92 (57.5)	78 (48.8)	
C	51 (31.9)	68 (42.5)	82 (51.2)	
Crude OR (95% CI) ^∗^	2.24 (1.56–3.22)	—	Reference	< 0.001
HWE *p* value	—	—	0.752	—

*Note:* Asterisk “ ^∗^” denotes crude OR (95% CI) corresponds to the primary allelic comparison (HCC vs. control). Detailed crude and adjusted ORs under different genetic models are presented in Table 3.

### 3.3. Correlation Between STAT3 rs1053004 Polymorphism and HCC Risk

Logistic regression analysis was employed to evaluate the association between the STAT3 rs1053004 variation and the risk of HCC utilizing multiple genetic models (Table 3). In the comparison between HCC cases and the control group, those with the TT genotype exhibited a significantly elevated risk of HCC compared with those with the CC genotype (adjusted OR = 4.26, 95% CI: 1.89–9.62, *p* < 0.001), after controlling for age, sex, smoking status, and cirrhosis. Conversely, people with the CT genotype exhibited a higher risk compared with those with the CC genotype; however, the difference lacked statistical significance (adjusted OR = 1.98, 95% CI: 0.92–4.28, *p* = 0.082). In accordance with the prevailing model (TT + CT vs. CC), persons with the T allele exhibited a significantly elevated risk of HCC compared with their counterparts (adjusted OR = 2.72, 95% CI: 1.35–5.47, *p* = 0.005). The age‐ and gender‐adjusted OR was 2.85, with a 95% confidence interval of 1.49–5.42 and a statistically significant *p* value (*p* < 0.001). The OR, adjusted for age and gender in the allelic model, was 2.23, with a 95% confidence interval of 1.46–3.41 and a statistically significant *p* value (*p* < 0.001). The TT genotype exhibited a significantly increased risk of HCC development in individuals with CHB (adjusted OR = 2.18, 95% CI: 1.03–4.62, *p* = 0.042) under the codominant model. In the recessive model, a statistically significant difference was seen concerning the genotype (adjusted OR = 1.85, 95% CI: 1.01–3.37, *p* = 0.046), but the dominant model did not demonstrate statistical significance (adjusted OR = 1.62, 95% CI: 0.81–3.25, *p* = 0.175). The T allele significantly influenced the risk of HCC in persons with CHB (adjusted OR = 1.57, 95% CI: 1.04–2.37, *p* = 0.032). Following numerous test changes, the significant results between the HCC and control groups remained, in contrast to those observed between HCC and CHB patients.

**Table 3:** Correlation between STAT3 rs1053004 polymorphism and hepatocellular carcinoma risk across various genetic models.

Comparison	Genetic model	Genotype	Crude OR (95% CI)	*p*	Adjusted OR∗(95% CI)	*p*
HCC versus control	Codominant	CC	1.00 (Reference)	—	1.00 (Reference)	—
		CT	1.79 (0.89–3.62)	0.104	1.98 (0.92–4.28)	0.082
		TT	4.29 (2.06–8.93)	< 0.001	4.26 (1.89–9.62)	< 0.001
	Dominant	CC	1.00 (Reference)	—	1.00 (Reference)	—
		CT + TT	2.67 (1.41–5.03)	0.002	2.72 (1.35–5.47)	0.005
	Recessive	CC + CT	1.00 (Reference)	—	1.00 (Reference)	—
		TT	2.86 (1.61–5.08)	< 0.001	2.85 (1.49–5.42)	0.001
	Allelic	C	1.00 (Reference)	—	1.00 (Reference)	—
		T	2.24 (1.56–3.22)	< 0.001	2.23 (1.46–3.41)	< 0.001
HCC versus CHB	Codominant	CC	1.00 (Reference)	—	1.00 (Reference)	—
		CT	1.38 (0.68–2.78)	0.372	1.37 (0.63–2.95)	0.427
		TT	2.23 (1.12–4.43)	0.022	2.18 (1.03–4.62)	0.042
		CC	1.00 (Reference)	—	1.00 (Reference)	—
		CT + TT	1.76 (0.93–3.34)	0.083	1.62 (0.81–3.25)	0.175
	Recessive	CC + CT	1.00 (Reference)	—	1.00 (Reference)	—
		TT	1.77 (1.02–3.06)	0.041	1.85 (1.01–3.37)	0.046
	Allelic	C	1.00 (Reference)	—	1.00 (Reference)	—
		T	1.58 (1.09–2.28)	0.016	1.57 (1.04–2.37)	0.032

*Note:* Asterisk “ ^∗^” denotes adjusted for age, sex, smoking status, and cirrhosis.

Abbreviations: CHB, chronic hepatitis B; CI, confidence interval; HCC, hepatocellular carcinoma; OR, odds ratio.

### 3.4. Stratified Analysis of STAT3 rs1053004 Polymorphism and Risk for Developing HCC

For the examination of the relationship between the STAT3 rs1053004 gene polymorphism and the increased risk of developing HCC, we performed a stratified analysis of the collected data depending on the presence/absence of the HBeAg, the amount of HBV DNA, and the presence/absence of cirrhosis (see Table 4). The relationship between the increased risk of developing HCC associated with the presence of the TT genotype proved to be much stronger in case of the absence of HBeAg (adjusted OR = 2.86, 95% CI = 1.37–5.97, *p* = 0.005) than in case of the presence of HBeAg (adjusted OR = 1.52, 95% CI = 0.65–3.57, *p* = 0.334). The correlation with the HBV DNA level was much stronger in case of high viral load (≥ 10^5^ IU/mL), providing adjusted OR of 3.14 (95% CI: 1.46–6.75, *p* = 0.003), in comparison with the low viral load (< 10^5^ IU/mL), that provided adjusted OR of 1.68 (95% CI: 0.77–3.66, *p* = 0.193). The correlation was markedly stronger in case of the presence of cirrhosis, providing adjusted OR of 2.97 (95% CI: 1.38–6.39, *p* = 0.005), than in case of the absence of cirrhosis, which provided adjusted OR of 1.43 (95% CI: 0.62–3.30, *p* = 0.406).

**Table 4:** Analysis of the relationship between the STAT3 rs1053004 genetic variant and the risk of developing hepatocellular carcinoma using a stratified approach.

Stratification	Subgroup	TT versus CT + CC adjusted OR ^∗^(95% CI)	*p*	*p* interaction
HBeAg status	Positive (*n* = 50)	1.52 (0.65–3.57)	0.334	0.041
	Negative (*n* = 110)	2.86 (1.37–5.97)	0.005	
HBV DNA level	≥ 10^5^ IU/mL (*n* = 87)	3.14 (1.46–6.75)	0.003	0.027
	< 10^5^ IU/mL (*n* = 73)	1.68 (0.77–3.66)	0.193	
Cirrhosis	Present (*n* = 89)	2.97 (1.38–6.39)	0.005	0.036
	Absent (*n* = 71)	1.43 (0.62–3.30)	0.406	

*Note:* Asterisk “ ^∗^” denotes adjusted for age, sex, and smoking status.

Abbreviations: CI, confidence interval; HBeAg, hepatitis B e antigen; HBV, hepatitis B virus; HCC, hepatocellular carcinoma; OR, odds ratio.

### 3.5. The Relationship Between the STAT3 rs1053004 Genetic Variation and Clinical Factors in HCC Patients

Association between STAT3 rs1053004 gene polymorphism and various clinical characteristic factors in patients with HCC is illustrated in Table 5. Median levels of AFP levels were significantly high in individuals with TT genotype (512.6 ng/mL) than CT genotype (285.3 ng/mL) and CC genotype (147.8 ng/mL) (*p* = 0.008). Moreover, individuals with TT genotype had significantly high HBV DNA levels (median: 8.3 × 10^5^ IU/mL) compared with CT genotype (3.6 × 10^5^ IU/mL) and CC genotype (1.9 × 10^5^ IU/mL) (*p* = 0.012). Advanced stages of BCLC (C/D) were significantly higher (56.4%) in patients with TT genotype than CT genotype (38.7%) and CC genotype (30.0%) (*p* = 0.031). No significant associations were found between various genotypes of STAT3 rs1053004 and tumor size, number, presence of tumor invasion, and Child–Pugh classification.

**Table 5:** Association between STAT3 rs1053004 genotypes and clinical parameters in HCC patients.

Parameter	TT (*n* = 39)	CT (*n* = 31)	CC (*n* = 10)	*p*
AFP (ng/mL), median (IQR)	512.6 (156.8–1857.3)	285.3 (84.2–942.5)	147.8 (53.6–583.9)	0.008
HBV DNA (IU/mL), median (IQR)	8.3 × 10^5^ (3.7 × 10^4^–1.6 × 10^7^)	3.6 × 10^5^ (1.8 × 10^4^–5.2 × 10^6^)	1.9 × 10^5^ (1.2 × 10^4^–2.8 × 10^6^)	0.012
Tumor size (cm), mean ± SD	5.7 ± 2.6	5.3 ± 2.4	4.9 ± 2.1	0.156
Tumor number, *n* (%)				0.283
Single	21 (53.8)	18 (58.1)	7 (70.0)	
Multiple	18 (46.2)	13 (41.9)	3 (30.0)	
Vascular invasion, *n* (%)	14 (35.9)	9 (29.0)	2 (20.0)	0.217
Child‐Pugh class, *n* (%)				0.374
A	13 (33.3)	12 (38.7)	4 (40.0)	
B	20 (51.3)	16 (51.6)	5 (50.0)	
C	6 (15.4)	3 (9.7)	1 (10.0)	
BCLC stage, *n* (%)				0.031
0/A	9 (23.1)	10 (32.3)	4 (40.0)	
B	8 (20.5)	9 (29.0)	3 (30.0)	
C/D	22 (56.4)	12 (38.7)	3 (30.0)	

Abbreviations: AFP, alpha‐fetoprotein; BCLC, Barcelona Clinic Liver Cancer; HBV, hepatitis B virus; HCC, hepatocellular carcinoma; IQR, interquartile range; SD, standard deviation.

## 4. Discussion

In this study, the relationship between STAT3 rs1053004 polymorphism and HCC development in the Iraqi population infected with chronic HBV has been examined. The results revealed that there is a strong relationship between T allele and TT genotype of STAT3 rs1053004 and high risk of HCC development in the Iraqi population. To the best of our knowledge, this is the first report that reveals the existence of a relationship between STAT3 rs1053004 and high risk of HCC in Iraqi population with chronic HBV infection. There was a relatively high frequency of T alleles observed in HCC patients in comparison with CHB patients and control subjects (48.8%; 68.1% in HCC and 57.5% in CHB cases). Besides, there was a 4.26‐fold increase found in the odds of having HCC in patients carrying the TT genotype of STAT3 rs1053004 compared with the CC genotype. More importantly, the abovementioned finding also holds true when comparing HCC patients and CHB patients, showing that the polymorphism of STAT3 rs1053004 could increase the susceptibility to HCC induced by HBV infection. Since the function mode of inheritance for the polymorphism has yet to be defined, several genetic models were examined in this investigation. Of all these, the recessive model remained significantly associated with HCC risk in the current study after taking into account other possible confounders. Nevertheless, further functional analysis is needed in order to validate if the genetic model indeed describes the functionality of the polymorphism. In the current investigation, our results are supported by other recent publications regarding STAT3 polymorphisms among different types of cancers. In one meta‐analysis conducted by Moazeni‐Roodi et al. [11], which included several case–control studies, the relationship between STAT3 gene polymorphisms and risk of developing cancer was studied. The results showed that the rs1053004 might affect the risk of developing cancer. However, due to the fact that no significant association was found through statistical analysis, the impact of the analyzed polymorphism remains unclear. Previous researches suggested that STAT3 polymorphisms might be related to the risk of developing HCC in patients infected with HBV. In the research conducted by Xie et al. [14], it was reported that the presence of rs1053004 might play an important role in the interactions between genetic and viral risk factors for HCC development. A stronger association identified in the current study (OR = 2.23, 95% CI: 1.46–3.41) compared with previous studies might depend on differences in genetic and viral profiles of the Iraqi population. Several recent studies conducted on samples from Iraq have revealed that the most frequent HBV genotype found in patients from this country is Genotype D, rather than B and C, which are the most frequent in East Asians [3]. In addition, it has been shown that the interaction of STAT3 polymorphisms with factors associated with HBV infection is an important determinant in HCC development [14]. At the same time, as was noted in [18], both patient genetics and viral factors play important roles in the pathogenesis of disease. Considering the well‐known link between STAT3 activation and inflammation and cancer growth [19], these influences can differ in different populations, depending on their genotype frequency. Our stratified analysis showed that the relationship between the studied polymorphism and HCC susceptibility varies according to some subgroups. Noting that one of the significant risk factors for HCC is liver cirrhosis, one must consider the large difference in cirrhosis prevalence in HCC versus CHB groups. Cirrhosis was considered as a covariate in multivariate logistic regression. However, even when adjustments were done, the relationship still showed significance, meaning that some level of confounding may not have been accounted for in this analysis, hence the need for a larger study population. The relationship seems stronger in patients who test negative for HBeAg and in those who have high viral load levels of greater than 10^5^ IU/mL, as well as in patients with cirrhosis. All of these seem to indicate that the impact of the STAT3 rs1053004 is mediated through gene–environment interaction, possibly due to the presence of both viral and/or clinical factors, and is supported by recent evidence on the importance of STAT3 in hepatocarcinogenesis caused by HBV infection [20]. The link found between the presence of TT genotype and high levels of AFP, high levels of HBV DNA, and the advanced BCLC stage could point to the possible contribution of rs1053004 polymorphism in HCC progression and development considering its association with STAT3, a well‐known player in tumor development [7, 21]. Specifically, the polymorphism rs1053004 is localized in the 3 ^′^‐UTR region of STAT3, which is believed to be important for posttranscriptional regulation of gene expression. Mutations in the 3 ^′^‐UTR regions affect miRNA binding and may result in altered expression of targeted genes, such as STAT3, leading to cancer development and progression [7, 22]. Additionally, the exploration of the regulatory annotations of rs1053004 polymorphism using RegulomeDB (Figure S4 and Table S5) revealed the presence of rs1053004 in the regulatory region that was confirmed through several lines of evidence, including chromatin accessibility, transcription factor binding (ChIP‐seq), and eQTL evidence. All the aforementioned statements, in their turn, suggest that this particular polymorphism may affect STAT3 expression regulation and serve as one more explanation to the found connection between STAT3 gene and HCC. In the present work, HWE analysis was mainly used as a quality control tool in the healthy control group, which showed no significant deviation from equilibrium, thereby confirming the reliability of the genotyping data.

However, at the same time, there are several important limitations that should be taken into account in regard to the presented results. First, the sample is rather small; therefore, the significance of the findings cannot be high. Second, taking into consideration the fact that the development of HCC is caused by different genetic factors working together, the investigation was conducted only for one particular SNP in the *STAT3* gene. In other words, it will be interesting to analyze how some of the STAT3 haplotypes can affect the probability of developing HCC. Third, it is important to conduct some functional experiments, as well as to clarify the effect of the identified SNP on *STAT3* gene activity in hepatocytes. Longitudinal studies conducted on patients with CHB infection with different STAT3 genotypes would help further establish the impact of these mutations on the development of HCC. A potential weakness of the study presented is the consideration of several genetic inheritance models, which might increase the risk of Type I errors. Despite that, the significant correlation seen between the STAT3 rs1053004 polymorphism and risk of HCC development remains the same when comparing both groups (HCC vs. healthy). However, some associations detected in the HCC–CHB comparison lost their statistical significance due to multiple testing correction and require further confirmation. Additional investigation with higher samples and replication using new populations is needed to validate results obtained. However, this study offers important information on the connection between the STAT3 rs1053004 mutation and HCC risk in HBV‐positive patients in Iraq. Considering the clinical relevance of this research, it can be stated that genotyping of the specified SNP would allow identifying patients at risk and assist in conducting screenings that would help detect HCC in a timely manner. The abovementioned results correlate with the contemporary guidelines for HCC screening among individuals with chronic liver disease and risk stratification [23]. The use of genetic markers in the specified context may improve existing screening procedures. Obtaining additional data about genetic factors contributing to the development of HCC would make it possible to develop personalized treatments. In practice, it is proven that taking into account genetic factors along with the other known ones helps to predict the probability of HCC occurrence [18]. Additionally, several researchers state that using genetic factors along with other screening tools, such as APRI, is necessary for HCC risk assessment, which is mentioned by Habeeb et al. [17].

## 5. Conclusion

This study concludes that there is a definite link between the STAT3 rs1053004 polymorphism and the risk of HCC among those who suffer from HBV infection. Specifically, those who carry the T allele, especially those with the TT genotype, are at a greater risk of developing the disease. It was also noted that these risks are mediated by various biological parameters such as HBeAg positivity, HBV DNA titer, and cirrhosis of the liver.

Further research using multicenter studies on larger samples should be conducted for validating the current results. Expanding the scope of investigation by considering other genetic factors, along with functional studies, will lead to more knowledge regarding STAT3 mutations and hepatocarcinogenesis.

## Author Contributions

Study concept and design: T.A.H. Data acquisition: H.A.A. and Z.Q.J. Data analysis and interpretation: H.A.A. Drafting of the manuscript: H.A.A. Critical revision of the manuscript for important intellectual content: T.A.H. and Z.F.S. Statistical analysis: Z.F.S. Obtained funding: none. Administrative, technical, or material support: Z.Q.J. Study supervision: T.A.H. Approval of final manuscript: H.A.A., T.A.H., Z.F.S., and Z.Q.J.

## Funding

No funding was received for this manuscript.

## Conflicts of Interest

The authors declare no conflicts of interest.

## Supporting information


**Supporting Information** Additional supporting information can be found online in the Supporting Information section. The supporting file includes additional figures (Figures S1–S6) and tables (Tables S1–S5) that provide detailed methodological information, bioinformatic analyses, additional statistical results, and supporting data related to the findings of this study.

## Data Availability

The data that support the findings of this study are available on request from the corresponding author. The data are not publicly available due to privacy or ethical restrictions.
