# Supplementary material for: Association of the STAT3 rs1053004 Polymorphism With Hepatocellular Carcinoma in Iraqi Patients With Hepatitis B
Source: Int J Hepatol. 2026 Jul 29;2026:6122232. doi: 10.1155/ijh/6122232 (PMC13416760; doi:10.1155/ijh/6122232)
Supplement: Supplementary file 1 — Supporting Information Additional supporting information can be found online in the Supporting Information section. The supporting file includes additional figures (Figures S1–S6) and tables (Tables S1–S5) that provide detailed methodological information, bioinformatic analyses, additional statistical results, and supporting data related to the findings of this study. [file IJH-2026-6122232-s001.docx]

**Supplementary Data for Validation and Functional Annotation of STAT3 rs1053004 Genotyping in HBV-Related Hepatocellular Carcinoma**

**Supplementary Table S1: Primer Sequences and Physicochemical Characteristics**

| Primer | Sequence (5′→3′) | Length (bp) | GC (%) | Tm (°C) |
| --- | --- | --- | --- | --- |
| T allele-specific Forward | TGGGAGTAGAGCTGGAGAT | 19 | 52.6 | 61.3 |
| C allele-specific Forward | TGGGAGTAGAGCTGGAGAC | 19 | 57.9 | 61.9 |
| Common Reverse | GCCACCTCCCTCTCCCTC | 18 | 72.2 | 65.1 |
| Internal Control Forward | CTGGGAGCAGAGCTGGAGA | 19 | 63.2 | 64.9 |

***The rs1053004 polymorphism is located within the 3′-UTR region of the STAT3 gene on chromosome 17 at genomic position chr17:42314074 (GRCh38.p14).*** ***Tm values were estimated using IDT OligoAnalyzer default conditions.***

**Supplementary Table S2: Amplicon Specificity and Expected Products**

| Amplicon | Primer Pair | Expected Size (bp) | Specificity |
| --- | --- | --- | --- |
| T allele product | T-specific Forward + Common Reverse | 120 | T allele |
| C allele product | C-specific Forward + Common Reverse | 120 | C allele |
| Internal control | Control Forward + Common Reverse | 220 | Quality-control amplicon |

***All amplicons were designed within the 3′-UTR region of the STAT3 gene.***

**Supplementary Table S3:Secondary Structure Analysis of ARMS-PCR Primers**

| Primer | Hairpin ΔG (kcal/mol) | Self-Dimer ΔG (kcal/mol) |
| --- | --- | --- |
| T allele-specific Forward | -0.13 | -6.34 |
| C allele-specific Forward | -0.13 | -6.34 |
| Common Reverse | Not detected | -3.14 |
| Internal Control Forward | -1.62 | -6.34 |

***Hetero-dimer analysis between the T allele-specific forward primer and the common reverse primer revealed a maximum ΔG value of −9.31 kcal/mol, indicating acceptable primer performance for PCR amplification.***

**Supplementary Table S4: In Silico Validation by Primer-BLAST**

| **Primer** | **Top BLAST Hit** | **Identity (%)** | **E-value** | **Specificity** | **Notes** |
| --- | --- | --- | --- | --- | --- |
| **Common Reverse** | Homo sapiens STAT3 gene, Chr17 | 100% | 8e-7 | Specific (single locus) | No off-target hits at ≥85% identity |
| **T-allele Specific Forward** | Homo sapiens STAT3 gene, Chr17 | 100% | 3e-8 | Specific (single locus) | Allele specificity was achieved through the 3′ terminal nucleotide corresponding to rs1053004. |
| **C-allele Specific Forward** | Homo sapiens STAT3 gene, Chr17 | 100% | 3e-8 | Specific (single locus) | Allele specificity was achieved through the 3′ terminal nucleotide corresponding to rs1053004. |
| **Control Forward** | Homo sapiens STAT3 gene, Chr17 | 100% | 4e-8 | Specific (single locus) | Yields 220 bp internal control amplicon |

***No significant off-target amplification was identified.***

**Supplementary Table S5: Functional Annotation of rs1053004**

| SNP | Gene | Location | RegulomeDB Rank | Regulatory Evidence |
| --- | --- | --- | --- | --- |
| rs1053004 | STAT3 | 3′-UTR | 1f | ChIP-seq, chromatin accessibility, and eQTL evidence |

**Supplementary Figure S1:Genomic Location of STAT3 rs1053004**

**
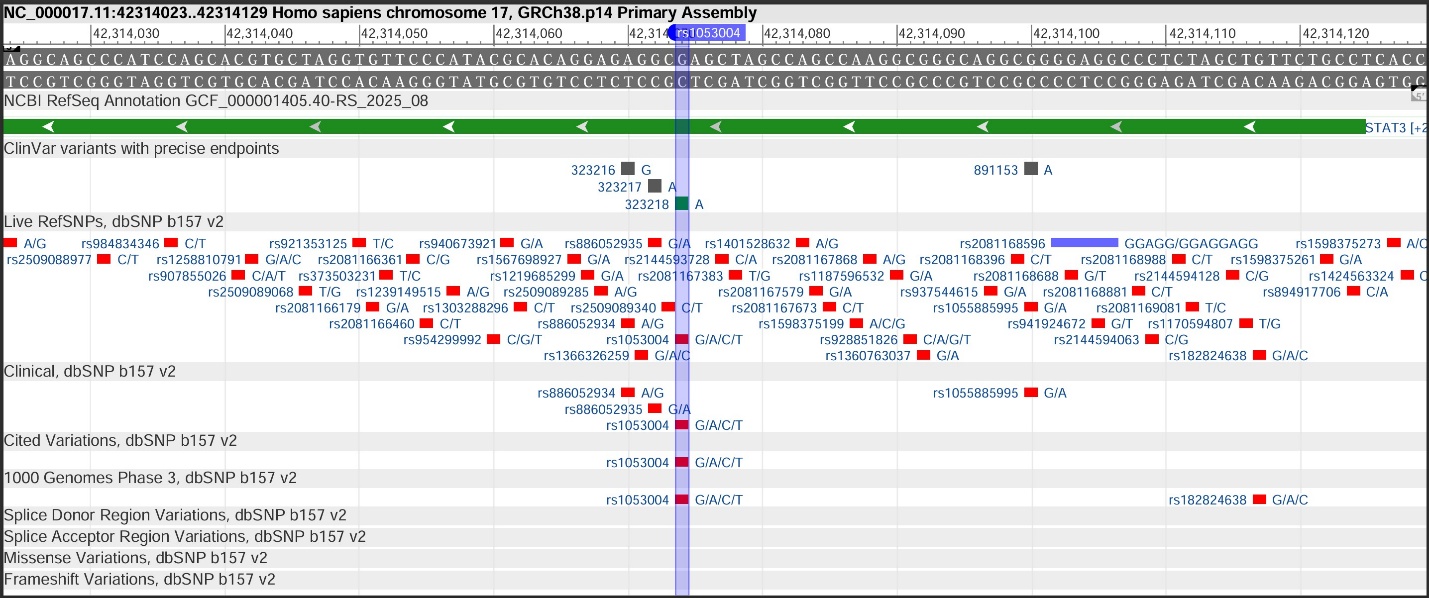
**

***Genomic view of the rs1053004 polymorphism obtained from the NCBI Variation Viewer (GRCh38.p14). The variant is located within the 3′-untranslated region (3′-UTR) of the STAT3 gene on chromosome 17 at genomic position chr17:42314074. The highlighted marker indicates the precise location of rs1053004 within the STAT3 locus.***

**Supplementary Figure S2:Design of allele-specific ARMS-PCR primers for genotyping of the STAT3 rs1053004 polymorphism.**

**
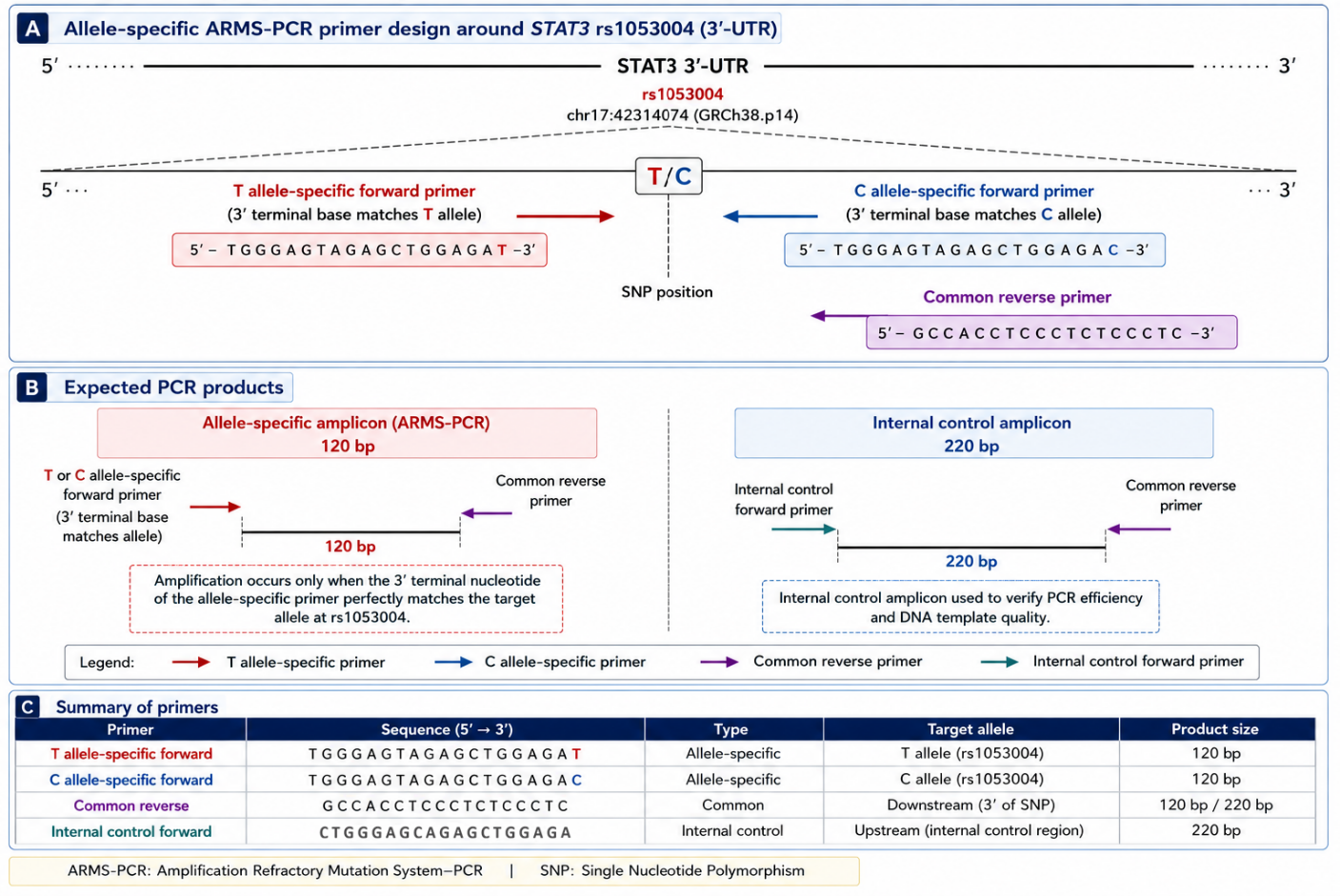
**
***Allele-specific forward primers targeting the T and C alleles of rs1053004 were used with a common reverse primer to generate a 120 bp allele-specific product. An internal control primer pair produced a 220 bp amplicon for assay validation.***

**Supplementary Figure S3:OligoAnalyzer Evaluation of ARMS-PCR Primers**


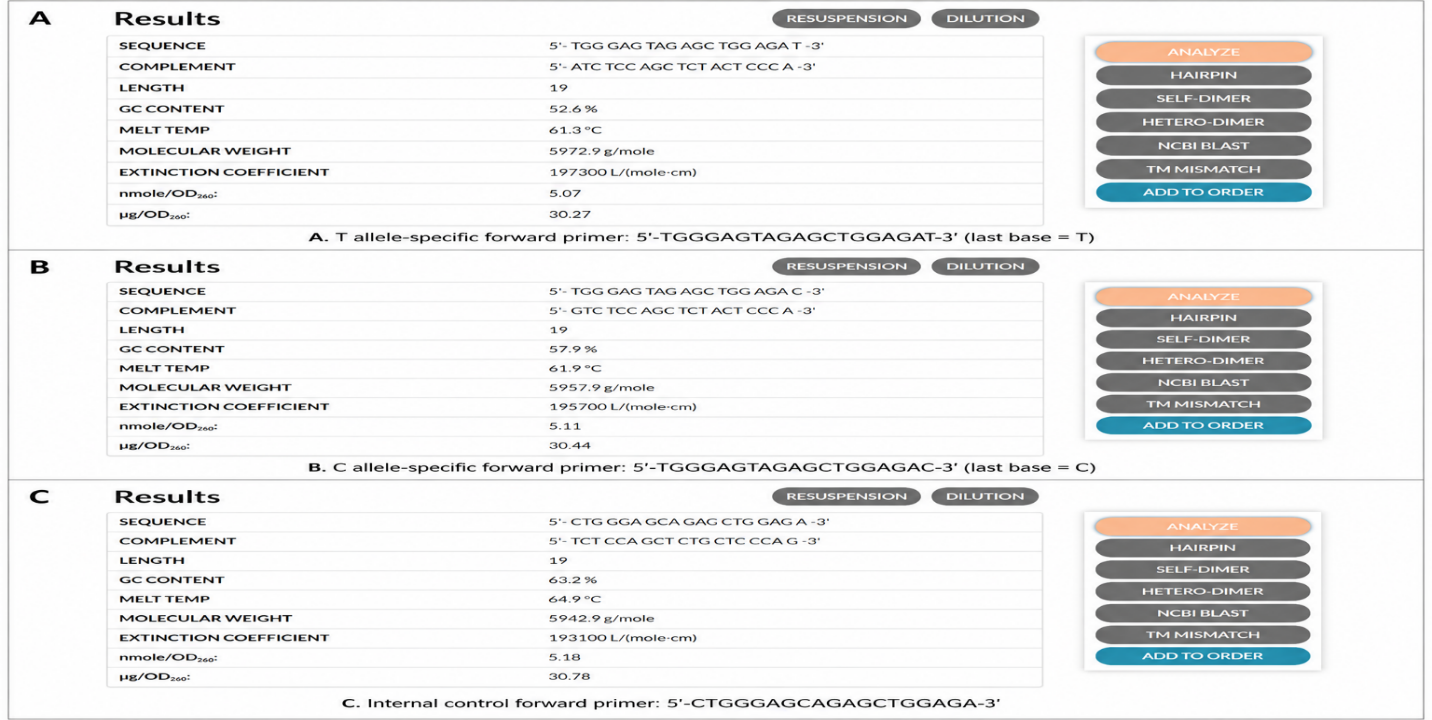


***OligoAnalyzer results showing primer sequence characteristics, GC content, melting temperature (Tm), molecular weight, hairpin formation, self-dimerization, and hetero-dimerization analyses. No significant secondary structures likely to interfere with amplification were identified.*** ***Hairpin, self-dimer, and hetero-dimer analyses demonstrated acceptable thermodynamic characteristics for ARMS-PCR amplification.***

**Supplementary Figure S4:Functional Annotation of rs1053004 Using RegulomeDB**


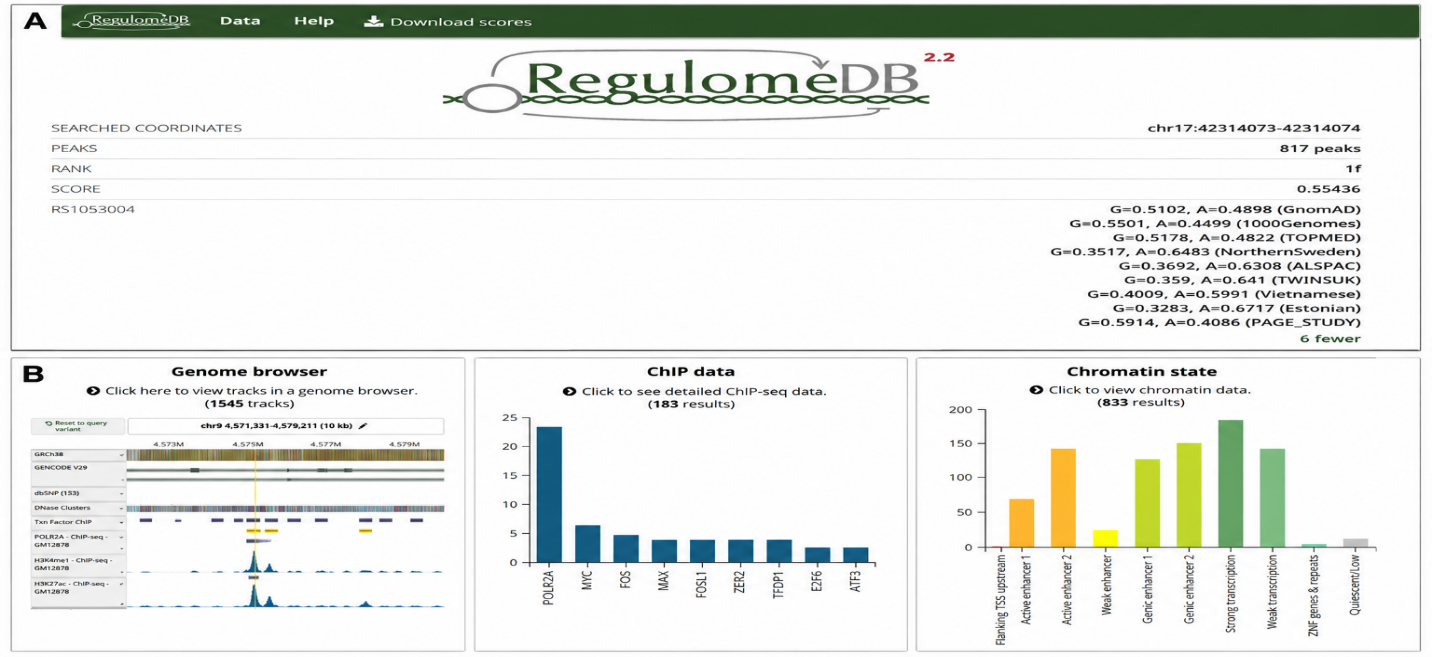


***RegulomeDB analysis of the STAT3 rs1053004 variant indicating evidence supporting a potential regulatory role of rs1053004 (A) RegulomeDB rank and score (Rank 1f) assigned to rs1053004. (B) Integrated data on the regulation of the variation, including chromatin accessibility, binding of the transcription factor through ChIP-seq studies and association with eQTL. These findings suggest that rs1053004 may influence gene regulation through effects on transcriptional activity and chromatin state.***

**Supplementary Figure S5. Distribution of continuous clinical variables across study groups.**


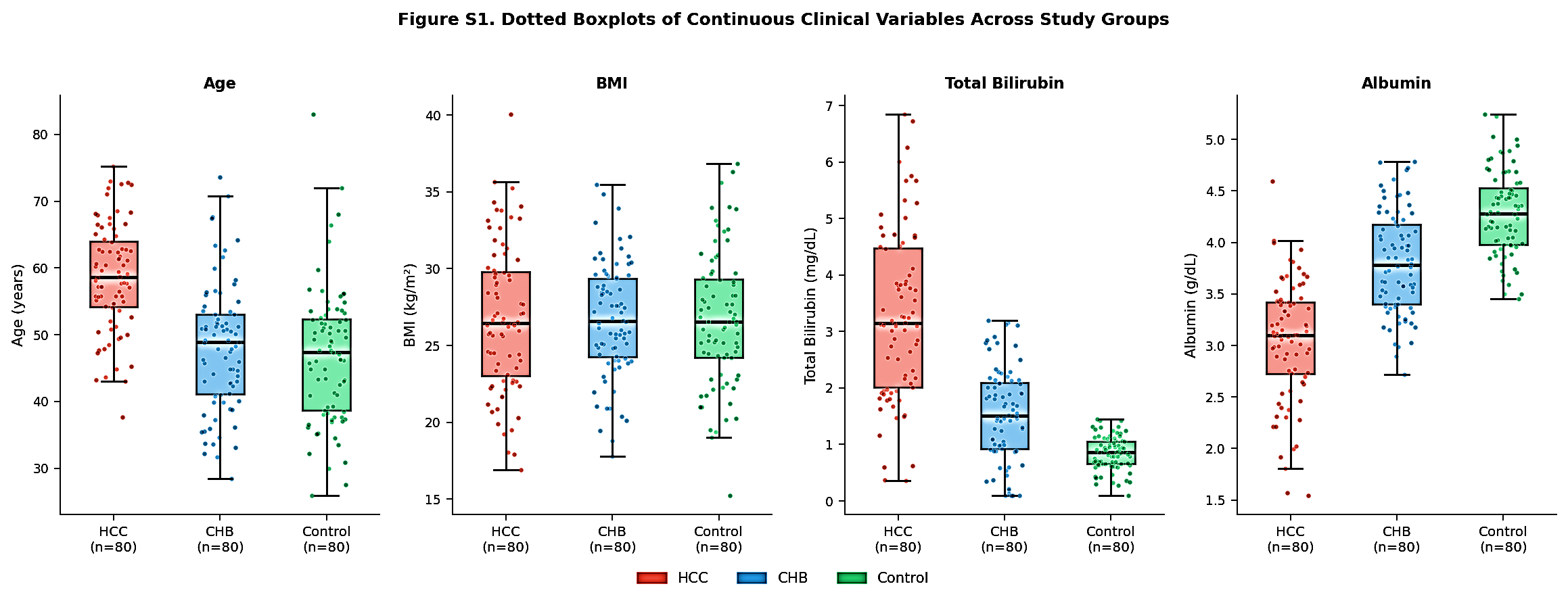


***Dotted boxplots showing distribution of continuous clinical variables across the three study groups. (A) Age (years); (B) Body mass index (BMI, kg/m²); (C) Total bilirubin (mg/dL); (D) Serum albumin (g/dL). Red: HCC group (n=80); Blue: CHB group (n=80); Green: Control group (n=80). Individual data points are overlaid as jittered dots. The horizontal line within each box represents the median; box boundaries represent the 25th and 75th percentiles (IQR); whiskers extend to 1.5×IQR. All P-values are as reported in Table 1 of the main manuscript.***

**Supplementary Figure S6. Distribution of serum biomarkers across study groups.**


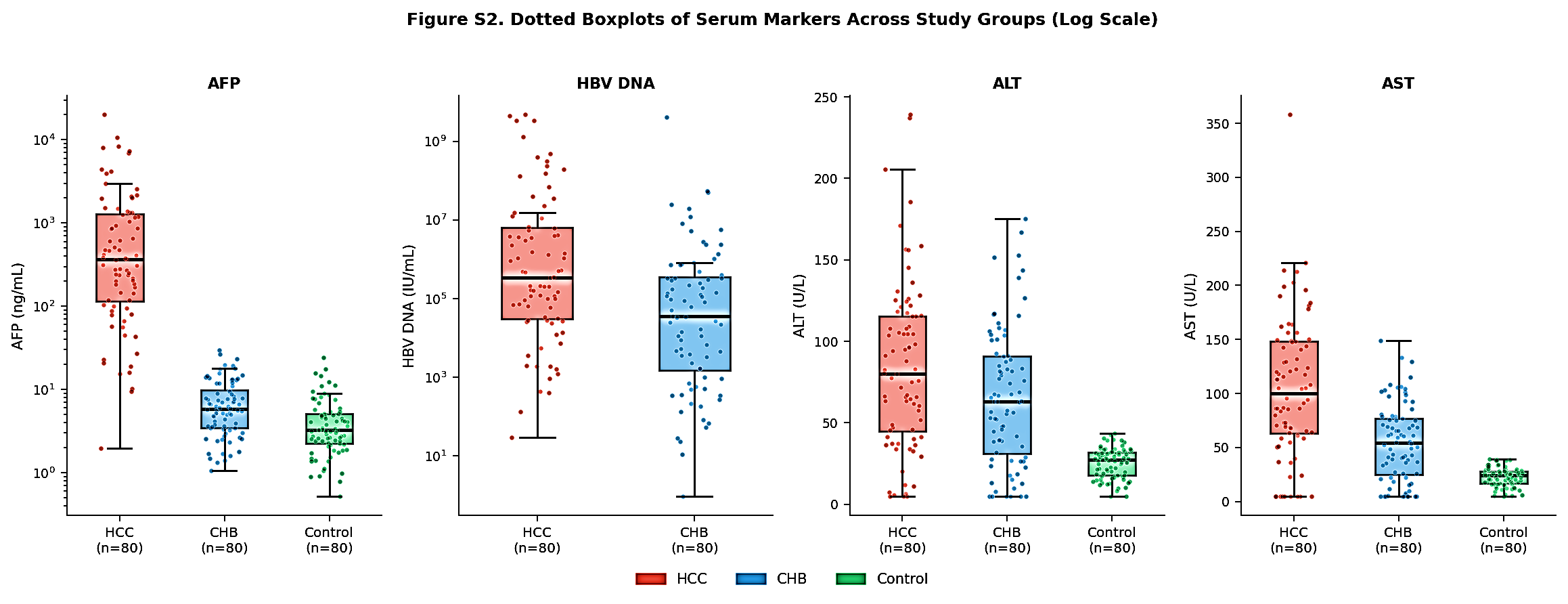


***Figure showing box plots of the distribution of serum biomarkers in the study groups where individual data points (jitter) are shown above the respective box plot*** ***Boxplots indicate the median, interquartile range (IQR; box) and whiskers to 1.5 times the IQR (A) Alpha-fetoprotein (AFP, ng/mL; log-scale); (B) HBV DNA (IU/mL; log-scale for HCC and CHB only); (C) Alanine aminotransferase (ALT, U/L); and (D) Aspartate aminotransferase (AST, U/L). Red indicates the HCC group (n = 80); blue shows the CHB group (n = 80); green indicates the healthy control group (n = 80). Statistical analysis and P-values have been included in Table 1 of the main paper.***

**Additional Statistical Details**

Variables expressed in mean ± standard deviation (SD) were compared using one-way ANOVA and followed by Bonferroni post hoc test. Variables presented as median and interquartile range were analyzed using the Kruskal-Wallis test, followed by Dunn’s multiple-comparison post hoc test. The comparison of HBV DNA between the HCC and CHB groups was conducted using the Mann-Whitney U test, as the healthy controls were intentionally HBV negative. Statistical analyses were conducted utilizing SPSS version 27.0 and R version 4.2.2.
